# Supplementary material for: HIVprotI: an integrated web based platform for prediction and design of HIV proteins inhibitors
Source: J Cheminform. 2018 Mar 9;10:12. doi: 10.1186/s13321-018-0266-y (PMC5845081; doi:10.1186/s13321-018-0266-y)
Supplement: Supplementary file 2 — Additional file 2. Source code of HIVProtI web server. [file 13321_2018_266_MOESM2_ESM.zip › HIVprotI_Source-code/hivprotI/weka-3-6-6/doc/weka/classifiers/evaluation/ConfusionMatrix.html]

ConfusionMatrix


---


|  |  |  |
| --- | --- | --- |
| **Overview** \| **Package** \| **Class** \| **Tree** \| **Deprecated** \| **Index** \| **Help** \| **Weka's home** | |  |
| PREV CLASS   **NEXT CLASS** | **FRAMES**    **NO FRAMES**     **All Classes** |  |
| SUMMARY: NESTED \| FIELD \| CONSTR \| METHOD | DETAIL: FIELD \| CONSTR \| METHOD |  |


---


## weka.classifiers.evaluation Class ConfusionMatrix

```
java.lang.Object
  weka.core.Matrix
      weka.classifiers.evaluation.ConfusionMatrix
```

**All Implemented Interfaces:**: java.io.Serializable, java.lang.Cloneable, RevisionHandler

---

``` public class ConfusionMatrix extends Matrix ```

Cells of this matrix correspond to counts of the number (or weight)
of predictions for each actual value / predicted value combination.

**Version:**
:   $Revision: 1.9 $

**Author:**
:   Len Trigg (len@reeltwo.com)

**See Also:**: Serialized Form

---

**Constructor Summary**

`ConfusionMatrix(java.lang.String[] classNames)`
  
          Creates the confusion matrix with the given class names.


| **Method Summary** | |
| --- | --- |
| `void` | `addPrediction(NominalPrediction pred)`             Includes a prediction in the confusion matrix. |
| `void` | `addPredictions(FastVector predictions)`             Includes a whole bunch of predictions in the confusion matrix. |
| `java.lang.String` | `className(int index)`             Gets the name of one of the classes. |
| `java.lang.Object` | `clone()`             Creates and returns a clone of this object. |
| `double` | `correct()`             Gets the number of correct classifications (that is, for which a correct prediction was made). |
| `double` | `errorRate()`             Returns the estimated error rate. |
| `java.lang.String` | `getRevision()`             Returns the revision string. |
| `TwoClassStats` | `getTwoClassStats(int classIndex)`             Gets the performance with respect to one of the classes as a TwoClassStats object. |
| `double` | `incorrect()`             Gets the number of incorrect classifications (that is, for which an incorrect prediction was made). |
| `ConfusionMatrix` | `makeWeighted(CostMatrix costs)`             Makes a copy of this ConfusionMatrix after applying the supplied CostMatrix to the cells. |
| `int` | `size()`             Gets the number of classes. |
| `java.lang.String` | `toString()`             Calls toString() with a default title. |
| `java.lang.String` | `toString(java.lang.String title)`             Outputs the performance statistics as a classification confusion matrix. |
| `double` | `total()`             Gets the number of predictions that were made (actually the sum of the weights of predictions where the class value was known). |

**Methods inherited from class weka.core.Matrix**

`add, addElement, eigenvalueDecomposition, getColumn, getElement, getL, getRow, getU, isSymmetric, LUDecomposition, main, multiply, numColumns, numRows, parseMatlab, regression, regression, setColumn, setElement, setRow, solve, toMatlab, transpose, write`

**Methods inherited from class java.lang.Object**

`equals, getClass, hashCode, notify, notifyAll, wait, wait, wait`

**Constructor Detail**

### ConfusionMatrix

```
public ConfusionMatrix(java.lang.String[] classNames)
```

:   Creates the confusion matrix with the given class names.

    **Parameters:**: `classNames` - an array containing the names the classes.


**Method Detail**

### makeWeighted

```
public ConfusionMatrix makeWeighted(CostMatrix costs)
                             throws java.lang.Exception
```

:   Makes a copy of this ConfusionMatrix after applying the
    supplied CostMatrix to the cells. The resulting ConfusionMatrix
    can be used to get cost-weighted statistics.

    :   **Parameters:**: `costs` - the CostMatrix. **Returns:**: a ConfusionMatrix that has had costs applied. **Throws:**: `java.lang.Exception` - if the CostMatrix is not of the same size as this ConfusionMatrix.

---


### clone

```
public java.lang.Object clone()
```

:   Creates and returns a clone of this object.

    :   **Overrides:**: `clone` in class `Matrix`
    :   **Returns:**: a clone of this instance.

---


### size

```
public int size()
```

:   Gets the number of classes.

    :   **Returns:**: the number of classes

---


### className

```
public java.lang.String className(int index)
```

:   Gets the name of one of the classes.

    :   **Parameters:**: `index` - the index of the class. **Returns:**: the class name.

---


### addPrediction

```
public void addPrediction(NominalPrediction pred)
                   throws java.lang.Exception
```

:   Includes a prediction in the confusion matrix.

    :   **Parameters:**: `pred` - the NominalPrediction to include **Throws:**: `java.lang.Exception` - if no valid prediction was made (i.e. unclassified).

---


### addPredictions

```
public void addPredictions(FastVector predictions)
                    throws java.lang.Exception
```

:   Includes a whole bunch of predictions in the confusion matrix.

    :   **Parameters:**: `predictions` - a FastVector containing the NominalPredictions to include **Throws:**: `java.lang.Exception` - if no valid prediction was made (i.e. unclassified).

---


### getTwoClassStats

```
public TwoClassStats getTwoClassStats(int classIndex)
```

:   Gets the performance with respect to one of the classes
    as a TwoClassStats object.

    :   **Parameters:**: `classIndex` - the index of the class of interest. **Returns:**: the generated TwoClassStats object.

---


### correct

```
public double correct()
```

:   Gets the number of correct classifications (that is, for which a
    correct prediction was made). (Actually the sum of the weights of
    these classifications)

    :   **Returns:**: the number of correct classifications

---


### incorrect

```
public double incorrect()
```

:   Gets the number of incorrect classifications (that is, for which an
    incorrect prediction was made). (Actually the sum of the weights of
    these classifications)

    :   **Returns:**: the number of incorrect classifications

---


### total

```
public double total()
```

:   Gets the number of predictions that were made
    (actually the sum of the weights of predictions where the
    class value was known).

    :   **Returns:**: the number of predictions with known class

---


### errorRate

```
public double errorRate()
```

:   Returns the estimated error rate.

    :   **Returns:**: the estimated error rate (between 0 and 1).

---


### toString

```
public java.lang.String toString()
```

:   Calls toString() with a default title.

    :   **Overrides:**: `toString` in class `Matrix`
    :   **Returns:**: the confusion matrix as a string

---


### toString

```
public java.lang.String toString(java.lang.String title)
```

:   Outputs the performance statistics as a classification confusion
    matrix. For each class value, shows the distribution of
    predicted class values.

    :   **Parameters:**: `title` - the title for the confusion matrix **Returns:**: the confusion matrix as a String

---


### getRevision

```
public java.lang.String getRevision()
```

:   Returns the revision string.

    :   **Specified by:**: `getRevision` in interface `RevisionHandler` **Overrides:**: `getRevision` in class `Matrix`
    :   **Returns:**: the revision


---


|  |  |  |
| --- | --- | --- |
| **Overview** \| **Package** \| **Class** \| **Tree** \| **Deprecated** \| **Index** \| **Help** \| **Weka's home** | |  |
| PREV CLASS   **NEXT CLASS** | **FRAMES**    **NO FRAMES**     **All Classes** |  |
| SUMMARY: NESTED \| FIELD \| CONSTR \| METHOD | DETAIL: FIELD \| CONSTR \| METHOD |  |


---
